# Involvement of DNA Damage Response via the Ccndbp1–Atm–Chk2 Pathway in Mice with Dextran-Sodium-Sulfate-Induced Colitis

**DOI:** 10.3390/jcm11133674

**Published:** 2022-06-25

**Authors:** Ryoko Horigome, Kenya Kamimura, Yusuke Niwa, Kohei Ogawa, Ken-Ichi Mizuno, Koichi Fujisawa, Naoki Yamamoto, Taro Takami, Tomoyuki Sugano, Akira Sakamaki, Hiroteru Kamimura, Masaaki Takamura, Shuji Terai

**Affiliations:** 1 Division of Gastroenterology and Hepatology, Graduate School of Medical and Dental Sciences, Niigata University, Niigata 951-8510, Japan; hrgm_ryonryon1127@nifty.com (R.H.); yusuke.28chan@gmail.com (Y.N.); kogawa@med.niigata-u.ac.jp (K.O.); kmizuno@med.niigata-u.ac.jp (K.-I.M.); t-sugano@med.niigata-u.ac.jp (T.S.); saka-a@med.niigata-u.ac.jp (A.S.); hiroteruk@med.niigata-u.ac.jp (H.K.); atmc@med.niigata-u.ac.jp (M.T.); terais@med.niigata-u.ac.jp (S.T.); 2 Department of General Medicine, Niigata University School of Medicine, Niigata 951-8510, Japan; 3 Department of Gastroenterology and Hepatology, Graduate School of Medicine, Yamaguchi University, Ube 753-8511, Yamaguchi, Japan; fujisawa@yamaguchi-u.ac.jp (K.F.); nao-yama@yamaguchi-u.ac.jp (N.Y.); t-takami@yamaguchi-u.ac.jp (T.T.)

**Keywords:** DSS, colitis, Ccndbp1, Atm, Chk2, DNA damage

## Abstract

The dextran sodium sulfate (DSS)-induced colitis mouse model has been widely utilized for human colitis research. While its mechanism involves a response to double-strand deoxyribonucleic acid (DNA) damage, ataxia telangiectasia mutated (Atm)–checkpoint kinase 2 (Chk2) pathway activation related to such response remains unreported. Recently, we reported that cyclin D1-binding protein 1 (*Ccndbp1*) activates the pathway reflecting DNA damage in its knockout mice. Thus, this study aimed to examine the contribution of *Ccndbp1* and the Atm–Chk2 pathway in DSS-induced colitis. We assessed the effect of DSS-induced colitis on colon length, disease activity index, and histological score and on the Atm–Chk2 pathway and the subsequent apoptosis in *Ccndbp1*-knockout mice. DSS-induced colitis showed distal colon-dominant Atm and Chk2 phosphorylation, increase in TdT-mediated dUTP-biotin nick end labeling and cleaved caspase 3-positive cells, and histological score increase, causing disease activity index elevation and colon length shortening. These changes were significantly ameliorated in *Ccndbp1*-knockout mice. In conclusion, *Ccndbp1* contributed to Atm–Chk2 pathway activation in the DSS-induced colitis mouse model, causing inflammation and apoptosis of mucosal cells in the colon.

## 1. Introduction

The dextran sodium sulfate (DSS)-induced colitis mouse model has been widely utilized in the research of human colitis, including ulcerative colitis (UC) [1]. UC is an inflammatory bowel disease that predominantly affects the distal colon [1]. Histologically, the colon in UC exhibits inflammatory cell infiltration, crypt damage, and ulceration, leading to colon length shortening [2]. These changes further cause diarrhea, rectal bleeding, and bodyweight loss, indicating a clinical disease activity in an inflammatory bowel disease such as UC [1,2]. In DSS-induced colitis, apoptosis occurs in the mucosal epithelia as a response to deoxyribonucleic acid (DNA) damage [3,4,5,6]. This response is caused by DSS damaging the oxidative DNA in the epithelia, inducing double-strand DNA breaks in the cells [7]. Generally, this double-strand DNA damage triggers ataxia telangiectasia mutated (Atm)–checkpoint kinase 2 (Chk2) pathway activation followed by p53-dependent apoptosis [8,9]. Atm-knockout mice show less apoptotic changes in the DSS-exposed colonic epithelium than wild-type mice [10]; however, the involvement of the Atm–Chk2 pathway in mouse colitis remains uninvestigated. Recently, we reported that cyclin D1-binding protein 1 (*Ccndbp1*)-knockout mice ameliorated the Atm–Chk2 pathway-related apoptosis induced by double-strand DNA damage [11]. Ccndbp1 (also known as GCIP and HHM) is a cyclin D-binding dominant-negative helix–loop–helix protein [12,13] expressed in various tissues, including the colon, small intestine, brain, thymus, lung, heart, spleen, liver, kidney, muscle, and peripheral leukocytes [14,15,16]. Therefore, we aimed to examine the contribution of *Ccndbp1* and the Atm–Chk2 pathway in DSS-induced colitis.

## 2. Materials and Methods

### 2.1. Animals

Animal experiments were approved by and conducted in full compliance with the regulations of the Institutional Animal Care and Use Committee at Niigata University in Niigata, Japan. Male BALB/c mice (8 weeks old, 25–30 g, *n* = 50) were purchased from CLEA Japan, Inc. (Tokyo, Japan). *Ccndbp1*-knockout mice (8 weeks old, 25–30 g, *n* = 50) were kindly provided by Yamaguchi University. Mice were housed in specific pathogen-free facilities under standard conditions at a temperature of 20–23 °C and humidity of 45–55% and fed with a standard diet.

### 2.2. DSS-Induced Colitis

DSS (MP Biomedicals, Irvine, CA, USA) was dissolved at 2% (*w/v*) in sterile drinking water and provided to mice ad libitum for one week. These mice were checked daily for colitis development by monitoring the disease activity index (DAI), which included body weight changes, stool consistency, and rectal bleeding. DAI was assessed according to a previously reported scoring system [17]: body weight loss: 0 = no loss, 1 = 1–5%, 2 = 5–10%, 3 = 10–20%, and 4 > 20%; stool consistency: 0 = normal, 2 = loose stool, and 4 = diarrhea; and rectal bleeding: 0 = negative for blood, 2 = slight bleeding, and 4 = gross bleeding. Hence, the maximum possible score was 12. Mice were sacrificed on day 5 and 8 after the initiation of DSS. The entire colon was removed to measure its length from the colon–cecal junction to the anal verge. Next, it was opened longitudinally, followed by blood confirmation. Thereafter, the colon was divided into two equal segments of the proximal and distal colon.

### 2.3. Histological Analysis

Each collected tissue was fixed in 10% formalin and embedded in paraffin. Five sections (5 µm) were then obtained from the tissue of transverse sections of the proximal and distal colon. Subsequently, standard hematoxylin and eosin staining and immunohistochemistry were performed. Histological score was assessed according to a previously reported scoring method [18]: severity of inflammation: 0 = rare inflammatory cells in the lamina propria, 1 = increased number of granulocytes in the lamina propria, 2 = confluence of inflammatory cells extending to the submucosa, and 3 = transmural extension of the inflammatory infiltrate; crypt damage: 0 = intact crypts, 1 = loss of the basal by one-third, 2 = loss of the basal by two-thirds, 3 = entire crypt loss, 4 = change in the epithelial surface with erosion, and 5 = confluent erosion; ulceration: 0 = no ulceration, 1 = 1 or 2 foci of ulcerations, 2 = 3 or 4 foci of ulcerations, and 3 = confluent or extensive ulceration. The maximum possible score was 11.

Immunohistochemical staining was conducted with a cleaved caspase 3 (Asp175) antibody (No. 9661; Cell Signaling Technology, Inc., Danvers, MA, USA) at 1:200 dilution; an anti-Atm antibody (ab78; Abcam, Cambridge, UK) at 1:200 dilution; an anti-phospho S1981 Atm antibody (ab36810; Abcam, Cambridge, UK) at 1:50 dilution using the Vectastain Elite ABC mouse IgG kit (PK-6102; Vector Laboratories, Burlingame, CA, USA); an anti-Chk2 antibody (No. 2662; Cell Signaling Technology, Inc., Danvers, MA, USA) at 1:100 dilution; an anti-phospho Thr68 Chk2 antibody (No. 2661; Cell Signaling Technology, Inc., Danvers, MA, USA) at 1:100 dilution; an anti-hepatocyte nuclear factor 4 alpha (HNF4α) (No. GTX54098; Gene Tex, Inc., Irvine, CA, USA) at 1:100 dilution using the Vectastain Elite ABC rabbit IgG kit (PK-6101; Vector Laboratories, Burlingame, CA, USA); and 3,3′-diaminobenzidine chromogen tablets (Muto Pure Chemicals, Tokyo, Japan). Images were captured for each tissue section randomly and quantitatively analyzed in ImageJ software (version 1.8.0_172; National Institutes of Health, Bethesda, MD, USA) with an RGB-based protocol, as reported previously [19], or by counting the number of positively stained cells.

### 2.4. Terminal-Deoxynucleotidyl-Transferase-Mediated Dutp-Biotin Nick End Labeling (TUNEL) Staining

Apoptotic cells were detected by TUNEL assay using the In Situ Apoptosis Detection kit (Takara Bio, Inc., Kusatsu, Shiga).

### 2.5. Whole-Transcriptome Sequencing

Whole-transcriptome sequencing of mice intestinal tissue was performed to investigate the different gene expression profiles and to perform gene annotation on a set of useful genes based on gene ontology pathway information (outsourced to DNAFORM, Yokohama, Kanagawa, Japan). Gene ontology (GO) enrichment analysis, which is based on the gene ontology (http://geneontology.org/, accessed on 1 January 2019) database, was conducted using the significant gene list in the g:Profiler tool (https://biit.cs.ut.ee/gprofiler/, accessed on 1 January 2019). The g:Profiler tool performs statistical enrichment analysis to determine over-representation of information from GO terms, biological pathways, regulatory DNA elements, human disease gene annotations, and protein–protein interaction networks, progressing to approximately three categories of GO. The gene or gene product associated with GO ID was summarized by parsing the ontology file and the annotation file (multispecies annotation provided by Uniprot or the annotation provided by each type reference DB for the GO consortium) for the GO graph structure. Each of the two tissue samples from the distal side of the colon of the animal groups were used for the analyses.

### 2.6. Reverse Transcription Polymerase Chain Reaction (RT-PCR)

The total RNA was extracted from the colon tissue of the DSS-treated wild-type mice or *Ccndbp1*-knockout mice using the RNeasy Mini kit (Qiagen, Hilden, Germany), reverse-transcribed into cDNA with the QuantiTect Reverse Transcription kit (Qiagen), and used for RT-PCR. Gene expression was measured by quantitative RT-PCR (qRT-PCR) using SYBR Green and the StepOnePlus System (Thermo Fisher Scientific, Inc., Waltham, MA, USA), and the results analyzed using bundled software. Thermal cycling conditions were as follows: 95 °C for 10 min, followed by 40 cycles of 94 °C for 15 s, 55 °C for 30 s, and 72 °C for 30 s, and melting hold at 95 °C for 15 s, 60 °C for 1 min, and 95 °C for 15 s. Changes in gene expression were calculated using the 2^−ΔΔCt^ method, with gene expression normalized to that of Gapdh for each sample. The primers used in this experiment were Gapdh, IFN-γ, IL-1β, IL-6, and IL-10 (Sigma–Aldrich, Tokyo, Japan).

### 2.7. Statistical Analyses

Data from each group are presented as the mean ± standard deviation (SD). Differences were evaluated by either one-way analysis of variance (ANOVA), followed by Bonferroni’s multiple comparison test, or Student’s t-test using GraphPad Prism9 software (version 9.3.1; GraphPad, San Diego, CA, USA). A *p* value of 0.05 or lower was considered statistically significant.

## 3. Results

### 3.1. Effect of DSS-Induced Colitis on Ccndbp1-Knockout Mice

**Figure 1:**
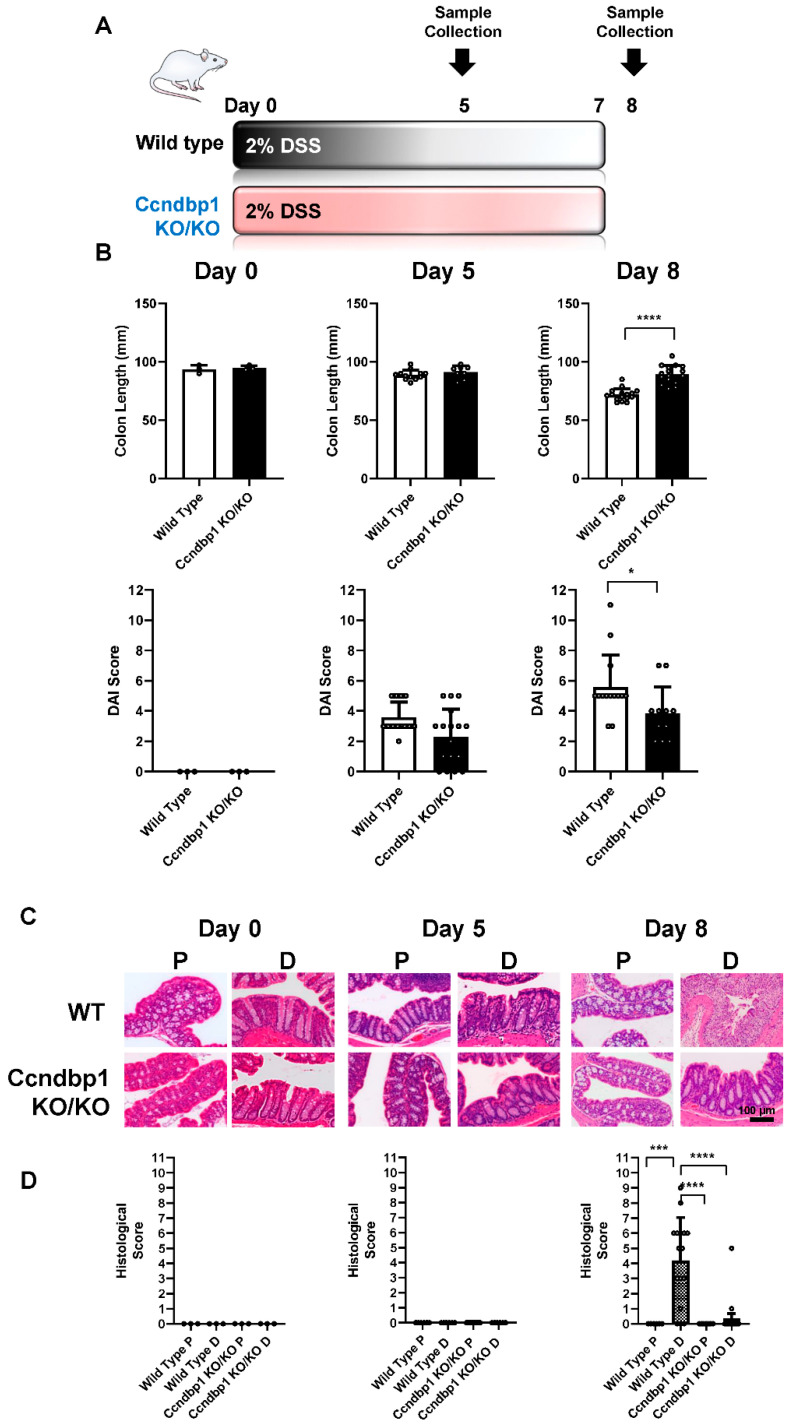
Effect of DSS-induced colitis on Ccndbp1-knockout mice. (**A**) Experimental design for dextran sodium sulfate (DSS)-induced colitis model mice (*n* = 8–10). (**B**) For each group, colon length and disease activity index (DAI) score were assessed on day 0, 5, and 8 after DSS initiation. DAI was assessed according to a previously reported scoring system, which includes the body weight loss, stool consistency, and rectal bleeding (maximum score: 12 points). The values represent the mean ± standard deviation (SD), * *p* < 0.05, Welch’s *t*-test. Each symbol represents the data of each mouse. (**C**) Representative images of hematoxylin and eosin staining of the colonic mucosa. Scale bar, 100 µm. (**D**) Time-dependent changes in the histological score, which includes the severity of inflammation, crypt damage, and ulceration (maximum score: 11 points). The values represent the mean ± standard deviation (SD) (*n* = 8–10), *** *p* < 0.001, **** *p* < 0.0001, one-way ANOVA with post hoc Tukey’s test. Each symbol represents the data of each mouse. WT: wild-type, P: proximal colon, D: distal colon.

To determine the role of Ccndbp1 and the Atm–Chk2 pathway in DSS-induced colitis, we examined the effect of DSS-induced colitis on Ccndbp1-knockout mice (Figure 1A). While the colon length of wild-type mice significantly shrank from 94.7 ± 2.1 mm to 71.8 ± 5.2 mm after one week of DSS administration, that of Ccndbp1-knockout mice remained unchanged, measuring 89.3 ± 7.8 mm, which was significantly longer than that of wild-type mice (Figure 1B). The DAI score, which included body weight changes, stool consistency, and rectal bleeding, increased time-dependently to 5.6 ± 2.1 points within one week in wild-type mice but was significantly suppressed in Ccndbp1-knockout mice (3.8 ± 1.8 points) (Figure 1B). According to the histological analyses of the colon (Figure 1C) and score in terms of the severity of inflammation (Figure 1D), DSS-induced colitis was more severe in the distal lesion (4.2 ± 2.9) than in the proximal lesion (0.0) of the wild-type mice on day 8, as previously reported [8]; however, no significant difference was seen in the Ccndbp1-knockout mice, reflecting milder inflammation in the entire colon (Figure 1). Thus, DSS-induced colitis in the epithelium was milder in Ccndbp1-knockout mice.

### 3.2. Effect of DSS-Induced Colitis on the Atm–Chk2 Pathway in the Colon

**Figure 2:**
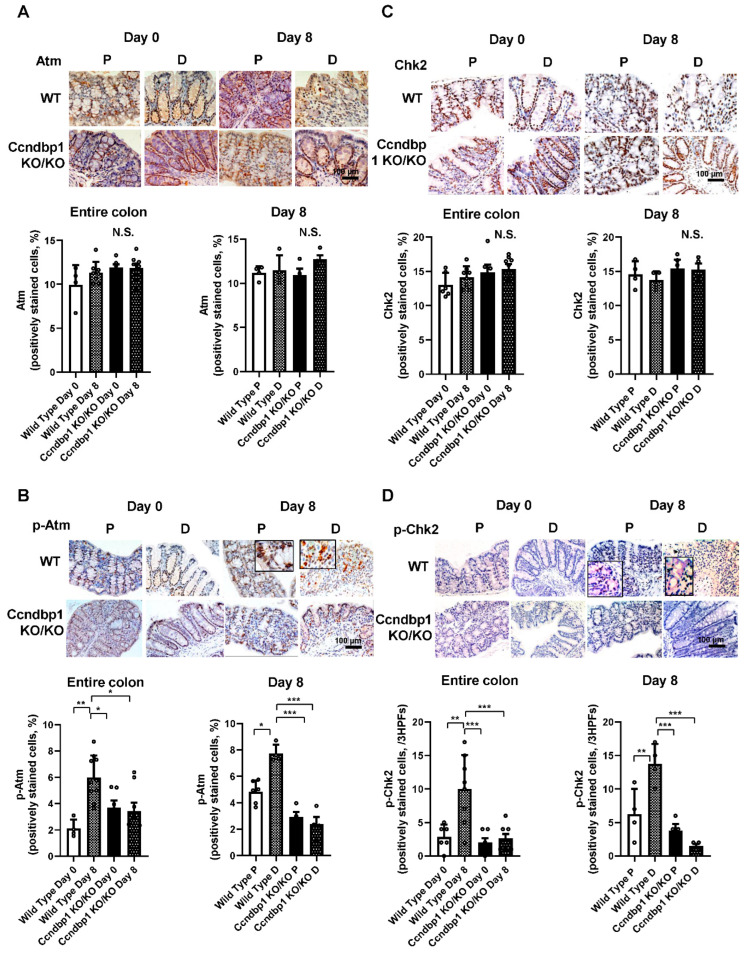
Expression of Atm, p-Atm, Chk2, and p-Chk2 in DSS-treated Ccndbp1-knockout mice. (**A**) Representative images of Atm expression in the colon of DSS-treated wild-type mice and Ccndbp1-knockout mice. Five different sections from each of the 5–6 mice in all groups were quantitatively analyzed for Atm expression assessment using ImageJ software for the entire colon and for the proximal or distal side of the colon. (**B**) Phospho-Atm (p-Atm), (**C**) Chk2, and (**D**) phospho-Chk2 (p-Chk2). Scale bar, 100 µm. The values represent the mean ± standard deviation (SD) (*n* = 5–6 mice per group), N.S., not significant, * *p* < 0.05, ** *p* < 0.01, *** *p* < 0.001, one-way ANOVA with post hoc Tukey’s test. Each symbol represents the mean data of each mouse. P: proximal colon, D: distal colon, HPFs: high-power fields.

Based on the results that Ccndbp1 depletion helped ameliorate DSS-induced colitis, the involvement of Atm–Chk2 pathway signaling in this disease was examined using the *Ccndbp1*-knockout mice. The Atm and Chk2 protein expression in the mucosal epithelia and their phosphorylation were assessed histologically (Figure 2A–D). Their expression (Figure 2A,C) showed no significant difference with or without DSS introduction between wild-type and *Ccndbp1*-knockout mice, and between proximal or distal lesions of the colon. However, their phosphorylation was significantly high in DSS-treated wild-type mice, and the ratio was higher in the distal colon than in the proximal colon (Figure 2B,D). In *Ccndbp1*-knockout mice, these phenomena were not seen, and the Atm–Chk2 pathway was not activated after DSS administration regardless of the colon lesion (Figure 2). Therefore, *Ccndbp1* contributed to activating the Atm–Chk2 pathway in DSS-induced colitis, as seen in the hepatocytes [11].

### 3.3. Effect of Ccndbp1 on DSS-Induced Apoptosis in the Colonic Mucosa

**Figure 3:**
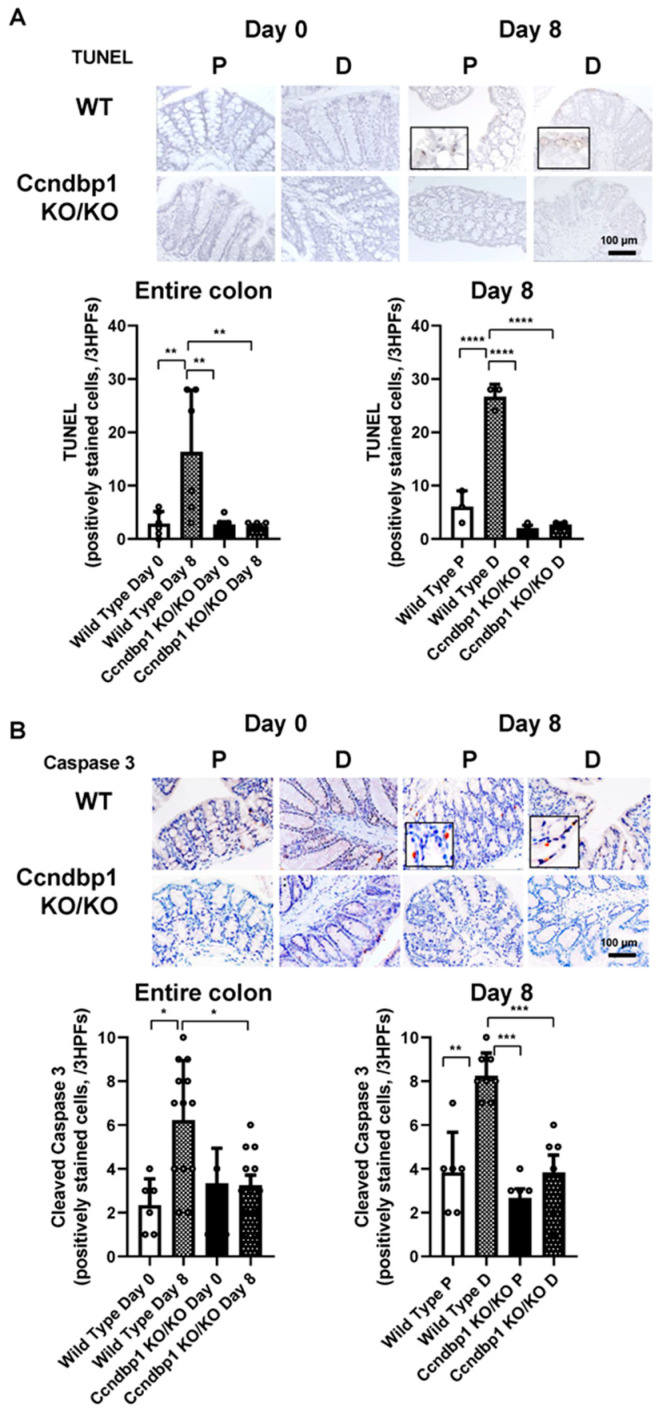
Expression of TUNEL and cleaved caspase 3 in DSS-treated colon samples. (**A**) Representative images of terminal deoxynucleotidyl transferase dUTP nick end labeling (TUNEL) staining in the colon of DSS-treated wild-type mice or Ccndbp1-knockout mice. Five different sections from each of the 5–6 mice in all groups were quantitatively analyzed for the entire colon and for the proximal or distal side of the colon. (**B**) Cleaved caspase 3. Scale bar, 100 µm. The values represent the mean ± standard deviation (SD) (*n* = 6–8 mice per group), * *p* < 0.05, ** *p* < 0.01, *** *p* < 0.001, **** *p* < 0.0001, one-way ANOVA with post hoc Tukey’s test. Each symbol represents the mean data of each mouse.

Given that *Ccndbp1*-knockout mice showed less inflammation and no Atm–Chk2 pathway activation by DSS-induced tissue damages, we examined mucosal apoptosis to determine if this milder inflammation was caused by the amelioration of the apoptosis. TUNEL staining showed that the positively stained cells in the distal colon of the wild-type mice significantly increased after DSS treatment, whereas no increase was seen in *Ccndbp1*-knockout mice (Figure 3A). This phenomenon was confirmed by the level of cleaved caspase 3, which reflects the earlier phase of apoptotic changes [20] in the colonic mucosal epithelial cells evidenced by HNF4α staining [20] (Figure 3B and Supplementary Figure S1A). Thus, *Ccndbp1* was involved in the damage response to DSS in the colonic mucosa via Atm–Chk2 pathway activation and apoptosis.

### 3.4. Effect of DSS on the Gene Expression in the Colon Tissue of Ccndbp1 KO/KO Mice

**Figure 4:**
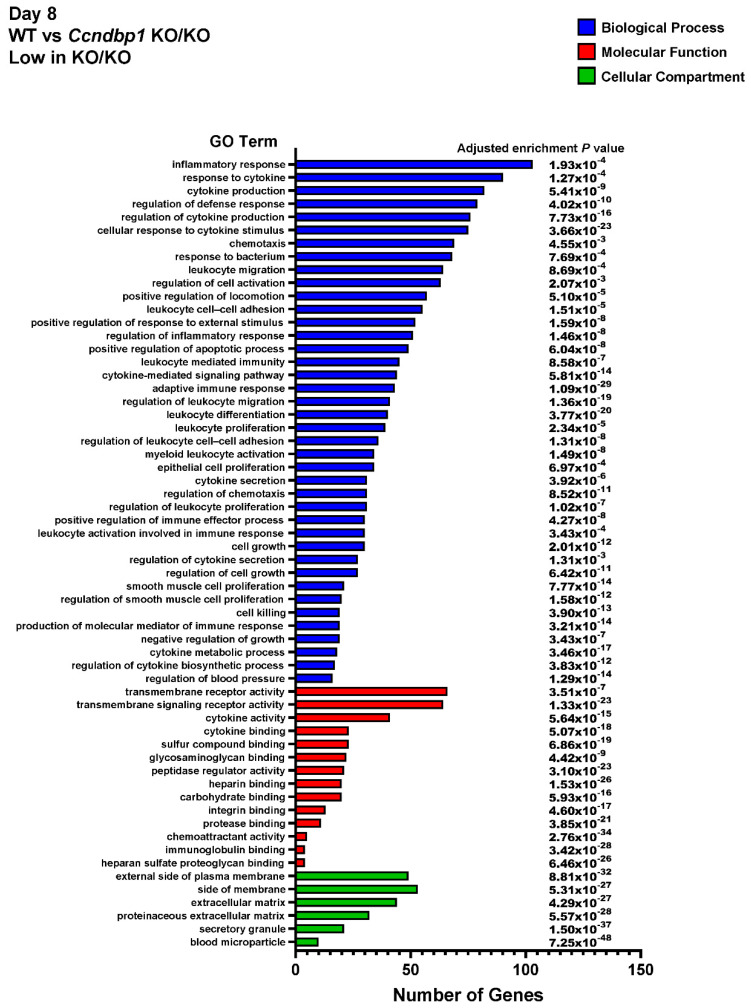
Gene Ontology (GO) enrichment analysis. Downregulated in Ccndbp1 KO/KO mice. Gene Ontology (GO) enrichment analysis of all the differentially expressed genes between DSS-treated wild-type mice or Ccndbp1-knockout mice on day 8 after DSS initiation. Vertical axis displays the number of significant genes corresponding to each functional type. All GO categories with an adjusted enrichment *p* value of less than 0.01 and more than 2-fold change are included. The categories of BP: biological process, MF: molecular function, and CC: cellular compartment are represented by blue, red, and green, respectively.

**Figure 5:**
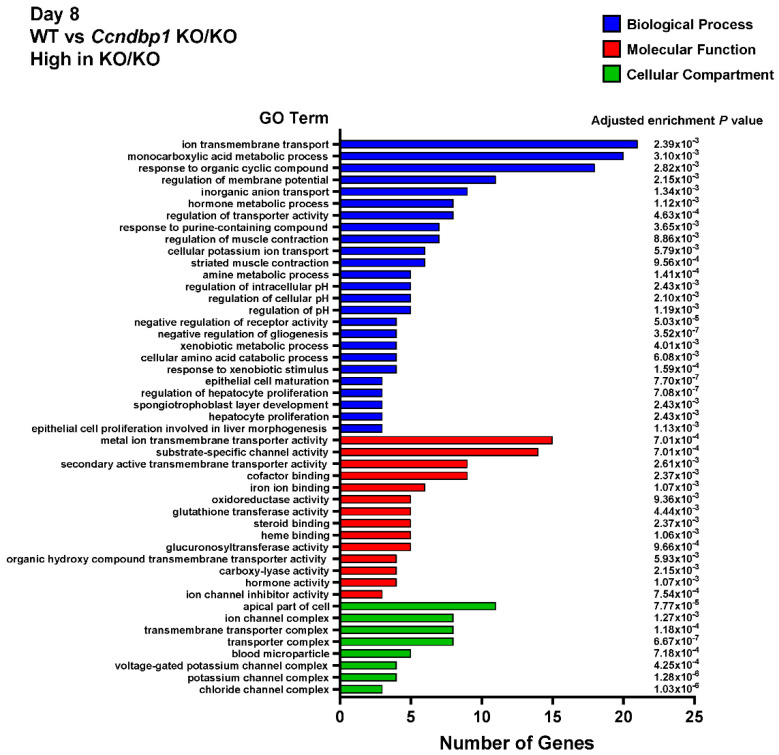
Gene Ontology (GO) enrichment analysis. Upregulated in Ccndbp1 KO/KO mice.

Then, the whole transcriptome sequencing assay was performed to examine the effect of DSS on the gene expression difference between wild-type and *Ccndbp1* KO/KO mice. The gene ontology enrichment analyses showed that genes related to the inflammatory response, response to cytokine, positive response to apoptosis process, etc., are lower (Figure 4) and genes related to transporter complexes are higher in Ccndbp1 KO/KO mice than wild-type mice (Figure 5). These results suggest that Ccndbp1 may also contribute on the inflammatory response after DSS damage on the mucosa.

## 4. Discussion

Our results demonstrated that *Ccndbp1* depletion ameliorated DSS-induced colitis in mice. The mucosa of *Ccndb1*-knockout mice exposed to DSS showed milder activation of the Atm–Chk2 pathway and apoptotic changes. These results are supported by a previous study focusing on the molecular mechanism of *Ccndbp1*. This study demonstrated that *Ccndbp1* overexpression contributes to resistance to Atm–Chk2 pathway activation that relies on double-strand DNA damage induced by X-ray [11]. Therefore, our study has revealed the molecular function of Ccndbp1 in the early phase of inflammatory changes following the direct DSS damage toward mucosa of the colon. This effect of DSS in the mice colitis model has previously been reported [7]. In addition, our results showed that *Ccndbp1* depletion contributed to the significantly lower level of inflammation-related gene expression (Figure 4) including, *IFN-γ*, *IL-1β*, *IL-6*, and *IL-10*, 8 days after the DSS initiation (Supplementary Figure S1B). These results indicated that Ccndbp1 might also be related to the chronic inflammation and inflammation-related carcinogenesis such as colitic cancer seen in ulcerative colitis [7]; therefore, further study focusing on the longer period of inflammation needs to be conducted. Our study results further showed that the colon length, histological inflammation, and DAI after DSS treatment were milder in *Ccndbp1*-knockout mice than in wild-type mice, consistent with the evidence that the pathology of DSS-induced colitis involves the double-strand DNA damage [7]. Therefore, our study is the first to report that *Ccndbp1* is involved in the pathogenesis of DSS-induced colitis in the acute phase via the Atm–Chk2 pathway and related apoptosis. The gene expression analysis including *Trp53* and caspases on day 8 after DSS initiation (Supplementary Figure S1C) also supported the results, and given that *Ccndbp1* is expressed in various organs, including the colon [14,15,16], based on the results obtained, it can be a therapeutic target for reducing the inflammation and apoptosis in colitis. In addition, considering that the DSS-induced colitis mouse model is the animal model for human colitis including UC [21,22], further analyses of the role of *Ccndbp1* in humans will also reveal the potential novel therapy that targets this molecule. However, apoptosis helps maintain biological homeostasis to eliminate potential malignant cells, which accumulates DNA damage and causes genomic instability [23]. Thus, further study is necessary to elucidate if Ccndp1 depletion or mutation is related to the occurrence of chronic inflammation assessing its role in the leukocytes [14,15,16] and colitic cancer in UC [22] conducting the longer-term study.

Our study has limitations. Firstly, *Ccndbp1* is related to the apoptosis and following inflammation; however, it is not directly related to the inflammatory cell recruitment as previously reported [11], even after DSS administration. These phenomena should further be validated with a molecular-based analysis of human colitis samples. In addition, although our recent study has proven that *Ccndbp1* can be a therapeutic target in hepatocellular carcinoma with poor response to conventional therapy [11], additional basic and clinical research is necessary to further gather important information using the specific inhibitors of the Atm–Chk2 pathway. Moreover, aside from the mucosal cell-based analyses, the role of inflammatory cells infiltrated in the mucosa should also be assessed.

## 5. Conclusions

In conclusion, *Ccndbp1* contributed to the activation of the Atm–Chk2 pathway in the DSS-induced colitis mouse model, triggering inflammation and apoptosis of mucosal cells in the colon.

## Supplementary Materials

The following supporting information can be downloaded at: https://www.mdpi.com/article/10.3390/jcm11133674/s1. Figure S1. (A) Representative images of HNF4 α staining in the colon of DSS-treated wild-type mice or Ccndbp1-knockout mice on day 8 after DSS initiation. (B) Gene expression related to the inflammation. Real-time polymerase chain reaction. (C) Gene expression related to the apoptosis and tight junctions determined by the ratio calculated based on the whole transcriptome sequencing.
