# Supplementary material for: Involvement of DNA Damage Response via the Ccndbp1–Atm–Chk2 Pathway in Mice with Dextran-Sodium-Sulfate-Induced Colitis
Source: J Clin Med. 2022 Jun 25;11(13):3674. doi: 10.3390/jcm11133674 (PMC9267230; doi:10.3390/jcm11133674)
Supplement: Supplementary file 1 [file jcm-11-03674-s001.zip › jcm-1731565-supplementary.pdf]

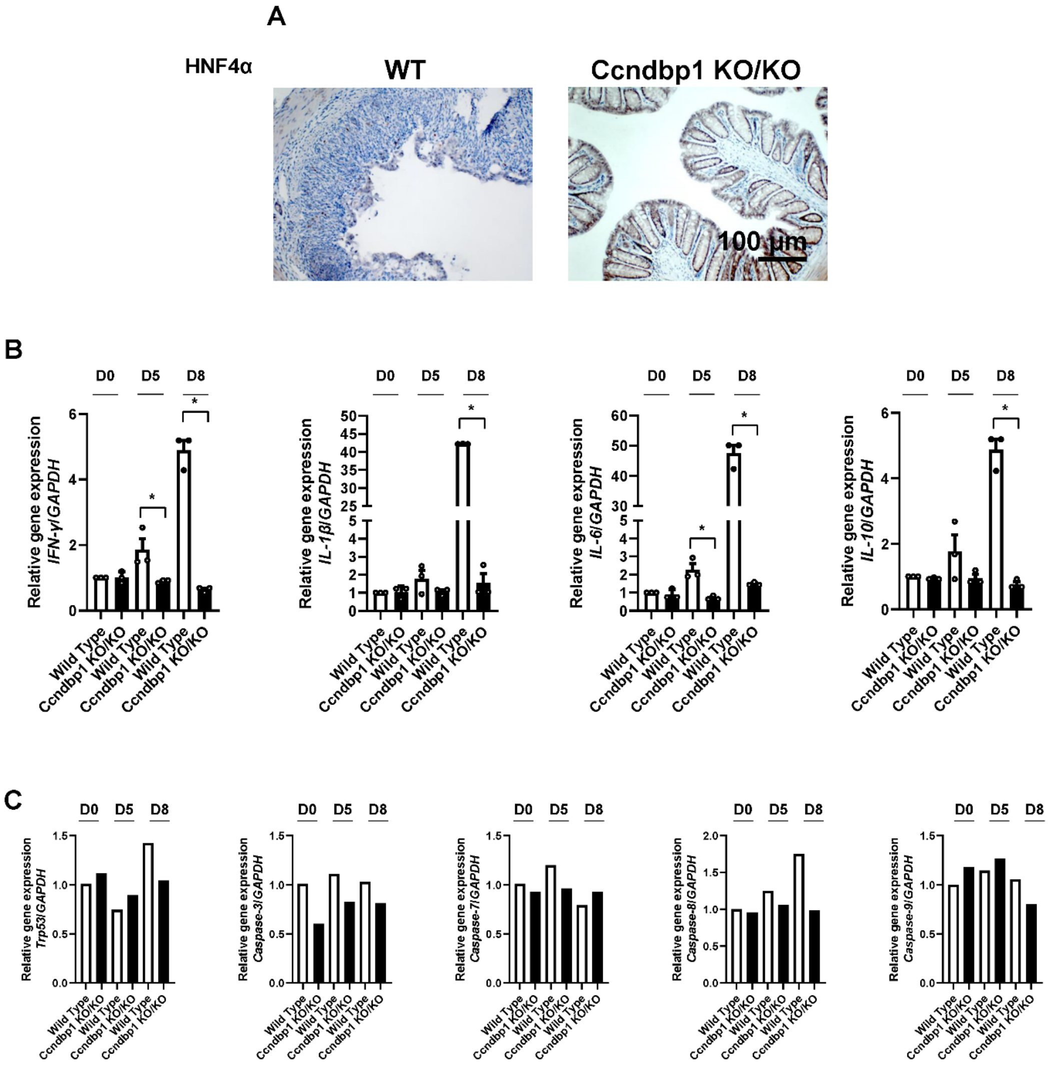


**Figure S1.** (A) Representative images of HNF4 $\alpha$ staining in the colon of DSS-treated wild-type mice or Ccndbp1-knockout mice on day 8 after DSS initiation. (B) Gene expression related to the inflammation. Real-time polymerase chain reaction. (C) Gene expression related to the apoptosis and tight junctions determined by the ratio calculated based on the whole transcriptome sequencing.
